# A proposed preventive role for Gamma-hydroxybutyrate (Xyrem^R^) in Alzheimer’s disease

**DOI:** 10.1186/s13195-016-0205-y

**Published:** 2016-09-06

**Authors:** Michel Maitre, Christian Klein, Ayikoe G. Mensah-Nyagan

**Affiliations:** Biopathologie de la Myéline, Neuroprotection et Stratégies Thérapeutiques, INSERM U1119, Fédération de Médecine Translationnelle de Strasbourg (FMTS), Université de Strasbourg, Bâtiment 3 de la Faculté de Médecine, 11 rue Humann, 67 000 Strasbourg, France

**Keywords:** Gamma-hydroxybutyrate, Xyrem^R^, Alzheimer’s disease, neuroprotection

## Abstract

Gamma-hydroxybutyrate (GHB or Xyrem^R^) is frequently used in humans for several clinical indications, including anesthesia, narcolepsy/cataplexy, and alcohol-withdrawal symptoms. Pharmacological effects induced in the brain by therapeutic doses of Xyrem^R^ are generally GABAergic-dependent. These effects allow sedation, stress/anxiety reduction, deep sleep induction, decrease of neuroinflammation, and neuroprotection. Furthermore, Xyrem^R^ promotes the expression of pivotal genes reducing toxic proteinopathies, as demonstrated in laboratory animal models. Altogether, these data represent additional evidence to suggest that Xyrem^R^ may be tested during repeated short periods in populations at risk for Alzheimer’s disease.

## Background

Gamma-hydroxybutyrate (GHB, sodium oxybate, or Xyrem^R^), which has long been approved by the U.S. Food and Drug Administration for the treatment of narcolepsy, cataplexy, or sleep disorders, is also commonly used in humans for anesthesia and the management of alcohol-withdrawal symptoms. Because GHB treatment efficiently improves neuropathological and cognitive symptoms in experimental or animal models of Alzheimer’s disease (AD), we review herein the essential positive effects of GHB supporting the possibility to use this drug in humans to prevent or delay the development of AD. Assessment of AD occurrence in aged narcoleptic patients treated with GHB compared to controls receiving another drug may also be an interesting strategy to check the therapeutic potential of GHB against AD. Thus, the present paper does not pretend to have the solution for the very complex AD issue. It must only be considered as a simple view suggesting a new option that will need a careful evaluation in selected groups of patients under strict medical supervision before any confirmation. Because AD remains a major health concern with no effective therapy available, every suggestion to prevent irreversible brain lesions or to improve patients’ lives needs to be considered with specific attention.

## Main text

Pre-clinical AD lasts several years or decades until the disrupted turnover and accumulation of Aβ in the brain reaches a critical level inducing the amyloid cascade. During this period, the activity of several critical proteins, regulated by multiple inherited or environmental factors, participates in the homeostasis of Aβ. It has recently been shown that GHB, administered at pharmacological dosages to rats or mice, modifies the brain transcriptome and induces several proteins exerting a beneficial role against proteinopathies, including AD [1, 2]. Among the genes upregulated by GHB, Neprilysin (NEP), Heat shock protein 70 (HSP70), and choline acetyltransferase (ChAT) are of deep interest for the management of the disease. Chronic administration of GHB to tg 2576 or phosphoramidon-treated mice reduced Aβ oligomers in the brain and normalized mnesic deficits compared to non-treated mice [3]. These beneficial effects were due to the ability of GHB to induce the expression of the endopeptidase NEP that potentiates the degradative clearance of Aβ oligomers. Therapeutic doses of GHB also promote HSP70, the ubiquitous class of ATP-dependent chaperone protein, which plays an important role in preventing protein misfolding and aggregation involved in AD pathogenesis [4]. Moreover, via the induction of ChAT, GHB could also improve the deficient cholinergic transmission [2].

Chronic GHB treatment may also have other targets of interest to delay or reduce AD, which is thought to be multi-factorial in about 95 % of patients. Interestingly, several pharmacological investigations revealed that the therapeutic doses of GHB evoke a large brain GABAergic potentiation which may subsequently generate the modulation of several other cellular components or factors [5]. Since GABA-A receptor agonists have shown neuroprotective effects against Aβ-induced neurotoxicity in animal and in vitro models, GHB-evoked GABAergic activation may be used at early stages to prevent the development of cognitive deficits [6]. At a metabolic point of view, the GABA shunt pathway has been proposed as an important source of energy in the situation of homeostasis failure, stress, hypoxia, and alteration of protein degradation. A part of exogenous GHB degradation is directly linked to the production of intermediates of this GABA shunt, which could be potentiated by the drug. In addition, it is generally thought that GHB reduces glucose oxidation and shifts the general energy metabolism in the direction of the phosphate shunt pathway [7]. This effect, added to the hyperactivity of NADP-dependent GHB dehydrogenase, contributes to the synthesis of NADPH and to the trapping of reactive oxidative agents, leading to the reduction of Aβ-induced vascular and neuronal stress. The possibility of GHB-induced oxidative stress was mentioned by an isolated in vitro study using rat tissue [8], but this question has never been documented in narcoleptic patients treated for many years with sodium oxybate (GHB) and the majority of these patients exhibited a good tolerability and efficacy for GHB [9]. The specific GABAergic profile of GHB at therapeutic doses could also possess beneficial properties on neuroinflammatory processes, which are key components of many neurodegenerative diseases, including AD. The brain level of GABA has been correlated to the progression of neuroinflammatory disorders [10]. In line with these data, the activation of GABA-A and GABA-B receptors decreased pro-inflammatory cytokines [10]. As GHB-related mechanism of action involves GABA-A and GABA-B receptors, GHB may exert anti-neuroinflammatory effects via the reduction of pro-inflammatory cytokines. However, appropriate experiments are required to check this hypothesis since the specific role of GHB on neuroinflammation has never been investigated.

Another property of GHB which may be interesting for AD prevention is its ability to induce sleep and to improve sleep architecture, particularly in promoting slow-wave sleep and delta power with a reduction of nocturnal awakening periods [11, 12]. There is growing evidence that sleep disturbances might be an early manifestation of AD and that the circadian rhythms are affected [13–15]. Whether sleep and/or biological rhythm-related problems may be causal factors or outcomes of neurodegenerative diseases remains a matter of speculation. However, noteworthy is the fact that the induction of sleep in a drosophila model of AD has successfully reversed memory defects [16]. In addition, it has been shown that the enhancement of GABAergic inhibition significantly rescued the slow-wave activity severely altered in a mouse model of AD [17]. Chronic sleep deprivation in this AD mouse model increased mitochondrial damages, caspase activation, and neuronal apoptosis in the hippocampus. These alterations were also observed in the wild-type littermates [18]. Mice that experienced sleep deprivation or an abnormal sleep-wake cycle had a significant increase in Aβ plaque pathology [15]. In humans, the non-REM sleep induced by a short period of sodium oxybate (GHB) treatment seems to be beneficial for the consolidation of declarative memory [19]. However, to get more valuable insights into the role of GHB against AD, it will be fundamental to compare, in narcoleptic populations, AD symptom occurrence in aged patients treated with GHB for several months/years versus control subjects treated with another drug. In case this comparative analysis confirms a beneficial action of GHB, people at risk (familial or environmental factors) for AD may be treated preventively with repeated periods of nocturnal GHB under medical supervision, at the onset of aging. Indeed, the appropriate protocols and dose regimens for this preventive GHB neuroprotective treatment will require a strict medical control taking into account the specific profile of each patient [20]. Hopefully, this strategy may help to prevent, reduce, or delay for several years the occurrence of irreversible brain lesions and AD symptoms.

It has been shown that GABAergic system dysfunction may contribute to cognitive impairment in humans and significant reductions in GABA levels have been described in severe cases of AD [21]. These decreases, beginning in middle age and impacting working memory, have been confirmed by magnetic resonance spectroscopy of the brain [22]. Additionally, a recent review quoted the existence of multiple alterations of the GABAergic neurotransmission in AD patients [23].

## Conclusions

GHB or Xyrem^R^ is generally a safe drug when given under medical supervision. Its genomic and pharmacological effects strongly support a preventive action of this drug to reduce the occurrence of cognitive deficits in selected patients at risk for AD (aging, familial, or genetic heritage). The drug is proposed to be given for a repeated period at night, in a similar manner to the strategy used for the treatment of narcolepsy. However, we do not suggest a chronic routine administration of GHB, but only short sequences of treatment (eight consecutive days every two months, for example, to be adapted following biological markers). These series of short periods of administration seem to be sufficient to reduce amyloid peptide accumulation, as demonstrated after NEP inhibition by phosphoramidon in mice [3]. In addition, we think that the proposed treatment should be delivered from the very early phase of the disease in order to increase the chance of effectiveness in preventing neurodegenerative processes. Again, and more importantly, we would like to recall that the present paper cannot warrant the success of the proposed treatment as it constitutes only an opinion or viewpoint, which lacks for the moment experimental data in humans. It should be mentioned that acute or sub-chronic use of the drug induces sometimes modest transient amnestic effects. Marked amnesia was reported following ingestion of illicit GHB, co-administered with antidepressants or ethanol, in recreative or criminal situations involving use/misuse of the drug. Moreover, as with many other drugs which are used daily in the human clinic (benzodiazepines, opiates, antidepressants, etc.), abuse uses of high concentrations of GHB may be addictive and/or may induce severe side effects (respiratory depression, coma, death). Therefore, the therapeutic use of GHB suggested herein may only be envisioned under strict medical supervision. In the authors’ opinion, the synthesis of more efficient compounds to replace GHB, especially in the domain of neuroprotection, has to focus on substances with long-lasting effects and strong agonistic activities for both GHB and GABA-B receptors [5].

## Abbreviations

AD: Alzheimer’s disease
ChAT: Choline acetyl-transferase
GABA: Gamma-aminobutyric acid
GHB: Gamma-hydroxybutyrate, sodium oxybate or Xyrem^R^
HSP70: Heat shock protein 70
NADP: nicotinamide adenine dinucleotide phosphate
NEP: Neprilysin
Tg 2576: Transgenic mice made which had the Swedish mutation integrated into the normal DNA of mice
